# Association of Characteristics of the Learning Environment and US Medical Student Burnout, Empathy, and Career Regret

**DOI:** 10.1001/jamanetworkopen.2021.19110

**Published:** 2021-08-09

**Authors:** Liselotte N. Dyrbye, Daniel Satele, Colin P. West

**Affiliations:** 1 Division of Community Internal Medicine, Department of Medicine, Mayo Clinic, Rochester, Minnesota; 2 Department of Health Sciences Research, Mayo Clinic, Rochester, Minnesota

## Abstract

**Importance:**

Previous studies have shown that medical student mistreatment and burnout are common. However, few longitudinal data exist to describe how mistreatment and other learning environment experiences are associated with subsequent burnout and other student characteristics.

**Objective:**

To examine the association between mistreatment and perceptions of the learning environment with subsequent burnout, empathy, and career regret among US medical students.

**Design, Setting, and Participants:**

This cohort study analyzed data from the 2014-2016 Association of American Medical Colleges (AAMC) Medical School Year 2 Questionnaire (Y2Q) and 2016-2018 AAMC Graduation Questionnaire (GQ). Medical students from 140 allopathic medical schools who responded to both AAMC surveys were included in the analysis. Data were analyzed from December 1, 2019, to January 11, 2021.

**Exposures:**

Self-reported medical student mistreatment (eg, experiences of negative behaviors and discrimination related to sex, race/ethnicity, and sexual orientation) and perceptions of the learning environment (Medical School Learning Environment Survey subscales for faculty, emotional climate, and student-student interactions).

**Main Outcomes and Measures:**

Burnout, empathy, and career regret as measured by Oldenburg Burnout Inventory data for burnout, Interpersonal Reactivity Index scores for empathy, and a single item assessing career regret.

**Results:**

Data from 14 126 medical students were analyzed; 52.0% were women, and the mean (SD) age was 27.7 (2.9) years at graduation. Mistreatment was reported by 22.9% of respondents on the Y2Q. In multivariable analysis adjusted for Y2Q measures, mistreatment reported on the Y2Q was associated with a higher exhaustion score (1.81 [95% CI, 1.60-2.02]), a higher disengagement score (0.71 [95% CI, 0.58-0.84]), and higher likelihood of career regret on the GQ (186 of 989 [18.8%]; all *P* < .001). A more positive emotional climate reported on the Y2Q was associated with a lower exhaustion score (for each 1-point increase, −0.05 [95% CI, −0.08 to −0.02]; *P* = .001) and lower disengagement score (for each 1-point increase, −0.04 [95% CI, −0.06 to −0.02]; *P* < .001) on the GQ. More positive faculty interactions on the Y2Q were associated with higher empathy score on the GQ (for each 1-point increase, 0.02 [95% CI, 0.01-0.05]; *P* = .04). Better student-student interactions were associated with lower odds of career regret during year 4 of medical school (odds ratio for each 1-point increase, 0.97 [95% CI, 0.95-1.00]; *P* = .04).

**Conclusions and Relevance:**

The findings of this cohort study suggest that medical students who experienced mistreatment and perceived the learning environment less favorably were more likely to develop higher levels of exhaustion and disengagement, lower levels of empathy, and career regret compared with medical students with more positive experiences. Strategies to improve student well-being, empathy, and experience should include approaches to eliminate mistreatment and improve the learning environment.

## Key Points

**Question:**

Is perceived mistreatment during medical school associated with burnout, empathy, and career regret at graduation?

**Findings:**

In this cohort study of 14 126 medical students, mistreatment by the beginning of year 2 was associated with severity of burnout and career regret toward the end of medical school, whereas positive experiences within the learning environment by the beginning of year 2 of medical school were associated with lower burnout, higher empathy, and less career regret during year 4.

**Meaning:**

These data imply that modifiable factors in the learning environment may contribute to burnout, empathetic orientation, and career satisfaction of medical students.

## Teaser

This cohort study assesses the association between learning environment experiences by the beginning of year 2 of medical school and subsequent reporting of burnout symptoms, empathy, and career regret during year 4 of medical school among respondents to the Association of American Medical Colleges Second-Year Survey and Graduation Questionnaire.

## Introduction

Medical students matriculate from undergraduate school with less burnout compared with peers who pursue other careers after college.^1^ Once in medical school, this narrative changes, with medical students more likely to experience burnout compared with similarly aged individuals in the population.^2^ In parallel, empathy declines for some students during medical training.^3,4,5,6^ Burnout or declines in empathy threaten professional identity formation, may negatively affect learning and patient care, and may influence specialty choice, increase suicidal ideation, and, in residents, lead to career regret.^7,8,9,10,11,12,13,14^

Studies have suggested that factors within the learning environment are associated with burnout, decline in empathy, and career regret among medical students and residents.^8,15,16^ Mistreatment, poor feedback, insufficient autonomy, high faculty demands, inadequate role models, and high workload are among the likely contributing factors.^6,8,17,18,19,20,21,22,23^ Few studies conducted to date, however, have been longitudinal or included a large national sample of trainees, limiting our understanding of the magnitude and direction of these associations.^8,24^ For example, we do not know if learners with burnout view the learning environment differently or if a poor learning environment increases the likelihood that learners experience burnout.^24,25^

To our knowledge, no previous longitudinal study has evaluated the association between the learning environment and subsequent burnout, empathy, or career regret among US medical students. Therefore, we obtained data from the responses of medical students to the Association of American Medical Colleges (AAMC) Medical School Year 2 Questionnaire (Y2Q) and Graduation Questionnaire (GQ) to explore the associations between learning environment experiences by the beginning of year 2 of medical school and subsequent reporting of burnout symptoms, empathy, and career regret during year 4 of medical school.

## Methods

We obtained deidentified medical student responses to the 2014-2016 AAMC Y2Q (administered early in year 2) and 2016-2018 AAMC GQ (administered toward the end of medical school). A unique numerical record identifier was used to link responses between the Y2Q and the GQ. The data set contained responses from 34 393 students to the Y2Q (10 307 of 20 348 for 2014; 11 625 of 20 624 for 2015; 12 461 of 20 947 for 2016; overall response rate, 55.5% [denominators are calculated based on the number of eligible second-year medical students at the time the survey closed; the actual number of eligible second-year medical students during the months the survey was open could have shifted slightly]) and responses from 47 078 students to the GQ (15 234 of 18 943 for 2016; 15 612 of 19 260 for 2017; 16 232 of 19 563 for 2018; overall response rate, 81.5%). Among these responders, 14 126 completed both the Y2Q and GQ and did not have missing Oldenburg Burnout Inventory (OBI) data. These 14 126 responders were included in this analysis. The final sample represented approximately 24.4% of allopathic US medical school graduates from 2016 through 2018. Data obtained included demographics (sex, age, marital status, and number of dependents) and career regret, along with measures of the learning environment, well-being, and empathy. The study was deemed exempt by the Mayo Clinic institutional review board, which waived the need for informed consent for the use of deidentified data. This cohort study followed the Strengthening the Reporting of Observational Studies in Epidemiology (STROBE) reporting guideline.

### Learning Environment

Measures of the learning environment on the Y2Q included items about social (relationships with others, including mistreatment, student-faculty relationships, and student-student relationships) and organizational (students’ affective response to educational experiences) experiences and perceptions. Consistent with previous studies of mistreatment of medical students,^26^ we included 16 items about personally experiencing negative behaviors (eg, public humiliation, unwanted sexual advances, threats or actual physical harm, bigoted remarks, and other offensive behaviors) and discrimination due to sex, race/ethnicity, and sexual orientation by faculty, nurses, resident/interns, other institutional employees or staff, or other students.^26^ Responders were asked to indicate how often they had experienced each of the behaviors during medical school using a 4-point scale (never, once, occasionally, and frequently). Responses were combined into a 3-point scale categorized into no mistreatment, mistreated once, and mistreated more than once. We also obtained students’ responses to the Medical School Learning Environment Survey (MSLES)^27^ on the Y2Q. The MSLES has 3 subscales: faculty interactions (Cronbach α = 0.79), emotional climate (Cronbach α = 0.92), and student-student interactions (Cronbach α = 0.79). Each subscale is calculated by summing across the items, which are measured on a 0- to 5-point scale (range, 0-20 for each subscale). Higher scores for each subscale indicate more positive perceptions of the learning environment.

### Well-being and Empathy

#### Burnout

The Y2Q and GQ surveys included a modified OBI to measure symptoms of burnout.^28^ Consistent with prior studies of medical students by Dahlin and colleagues,^29,30^ the word *work* was replaced with *studies*. In the AAMC questionnaires, the OBI items were further modified to better reflect exhaustion and disengagement from medical school studies. The original OBI response options were used across a 4-point Likert scale from strongly agree (0) to strongly disagree (3). Due to limited validity data in medical students, we randomly divided the Y2Q and GQ responders into development and validation cohorts and conducted exploratory factor analysis on each development cohort, followed by confirmatory factor analysis in the validation cohort (detailed results are provided in the eTable in the Supplement). As a result, we decided to include 8 items in the exhaustion subscale and 5 items in the disengagement subscale for both the Y2Q and GQ because these items provided the best fit at the GQ time (dependent variable). The resulting OBI exhaustion subscale had a Cronbach α of 0.81 for Y2Q and 0.83 for GQ, and the disengagement subscale had a Cronbach α of 0.77 for Y2Q and 0.72 for GQ. The possible range of scores was 0 to 24 for the exhaustion subscale and 0 to 15 for the personalization subscale, with higher scores indicating higher levels of exhaustion and disengagement.

#### Empathy, Quality of Life, and Stress

Empathy was measured using 4 items from each of the perspective taking and empathic concern subscales of the Interpersonal Reactivity Index (IRI).^31,32^ The IRI scores were calculated by summing across the 8 items, which were measured on a 0- to 4-point scale. The range of possible scores is 0 to 32, and higher scores indicate higher levels of empathy. The Cronbach α for the empathy measure was 0.77 for both Y2Q and GQ.

To control for concurrent overall quality of life (QOL) and stress, we included the single-item linear analogue QOL scale and the Perceived Stress Scale from the Y2Q. The single-item linear analogue QOL scale is widely used and has substantial validity data.^33,34,35,36,37,38^ Responders rate their overall QOL on a 10-point scale, with higher scores indicating better QOL. The 4-item Perceived Stress Scale has a 0- to 4-point response scale (range, 0-16).^39,40^ Higher scores indicate higher perceived levels of stress.

### Career Regret and Plans

Career regret was assessed on the Y2Q and GQ with an item used in previous studies of physicians.^23,41,42^ Participants were considered to have career regret if they responded “no” or “probably not” to the item “If you could revisit your career choice, would you choose to become a physician again?”

### Statistical Analysis

Data were analyzed from December 1, 2019, to January 11, 2021. We calculated descriptive summary statistics. We examined for differences in mistreatment and perceptions of the learning environment by burnout (exhaustion and disengagement), empathy, and career regret, adjusting for age, sex, relationship status, and parental status using a Wilcoxon, Mann-Whitney, or Fisher exact test as appropriate. All tests were 2-sided with a type I error of .05. We performed multiple linear or logistic regression analysis to evaluate associations of the independent variables, measured at the beginning of year 2 of medical school, with exhaustion, disengagement, empathy, and career regret, measured during year 4 of medical school. All models included mistreatment, MSLES subscale scores, OBI exhaustion and/or disengagement scores, IRI score, QOL score, Perceived Stress Scale score, and demographics (sex, age, marital status, relationship status, and number of dependents) as measured at the beginning of year 2 of medical school. The generalized linear regression model for career regret during year 4 of medical school also included career regret at the beginning of year 2 of medical school as an independent variable. All comparisons were performed using SAS, version 9.4 (SAS Institute Inc); *P* < .05 indicated statistical significance.

## Results

Among the 14 126 medical students in the cohort, 52.0% were women and 48.0% were men; the mean (SD) age was 27.7 (2.9) years; 72.8% were single; and 91.0% did not have dependents (Table 1). The sex distribution in our cohort, as reported in year 4 of medical school, was similar to the sex distribution of US medical students in corresponding graduating years.^43^

**Table 1.** Characteristics of Study Population as Reported on the AAMC GQ^a^

Characteristic	Values^b^
Sex	
Female	7348 (52.0)
Male	6777 (48.0)
No. missing	1
Age, mean (SD), y	27.7 (2.9)
Age range, y	
<24	33 (0.2)
24-26	5943 (42.1)
27-29	5766 (40.8)
30-32	1545 (10.9)
>32	839 (5.9)
Marital status	
Single	10 206 (72.8)
Legally married	3582 (25.6)
Common law or civil union	56 (0.4)
Divorced	137 (1.0)
Separated, but still legally married	26 (0.2)
Widowed	3 (0.02)
No. missing	116
No. of dependents	
0	12 744 (91.0)
1	730 (5.2)
≥2	533 (3.8)
No. missing	119

Abbreviations: AAMC, American Association of Medical Colleges; GQ, Graduation Questionnaire.

^a^ Includes 14 126 medical students who responded to the 2014, 2015, or 2016 AAMC Medical School Year 2 Questionnaire and the 2016, 2017, or 2018 AAMC GQ.

^b^ Unless otherwise indicated, data are expressed as No. (%) of patients, with denominators excluding the number missing.

With respect to burnout, the mean (SD) exhaustion scores reported on the Y2Q and GQ were 12.4 (4.0) and 12.4 (4.1), respectively (95% CI for difference, −0.12 to 0.01; *P* = .10). The mean (SD) disengagement scores reported on the Y2Q and GQ were 5.6 (2.5) and 5.4 (2.5), respectively (95% CI for difference, −0.32 to −0.24; *P* < .001). The mean (SD) empathy scores reported on the Y2Q and GQ were 21.0 (3.9) and 21.2 (3.9), respectively (95% CI for difference, 0.11-0.22; *P* < .001). In terms of career regret, the prevalence of medical students reporting that they would definitely not or probably not choose to become a physician again if given the chance to revisit their career choice increased from 563 of 14 123 (4.0%) on the Y2Q to 989 of 14 086 (7.0%) on the GQ (*P* < .001).

Slightly more than three-quarters (10 852 of 14 076 [77.1%]) of students reported never having been mistreated, whereas 1596 of 14 076 (11.3%) reported having been mistreated once and 1628 of 14 076 (11.6%) reported having been mistreated more than once on the Y2Q (total, 3224 of 14 076 [22.9%]). The mean (SD) MSLES scores reported on the Y2Q were 14.9 (0.8) for faculty interactions, 9.4 (3.1) for the emotional climate, and 15.2 (3.0) for student-student interactions.

Reported exhaustion, disengagement, empathy, and career regret on the GQ are shown in Table 2 by experience of mistreatment and perceptions of learning reported on the Y2Q. More frequent experience of mistreatment reported on the Y2Q was associated with higher levels of exhaustion (mean scores, 12.0 for never, 13.0 for once, and 13.8 for more than once) and disengagement (mean scores, 5.3 for never, 5.5 for once, and 6.0 for more than once) reported on the GQ (both *P* < .001) (Figure, A and B). Students who reported more frequent mistreatment on the Y2Q were also more likely to report career choice regret on the GQ (6.4% for never, 7.0% for once, and 11.4% for more than once; *P* < .001) (Figure, C).

**Table 2.** Association of Mistreatment and Perceptions of the Learning Environment Among US Second-Year Medical Students Reported on the AAMC Y2Q Survey With Subsequent Burnout and Empathy Scores and Reported Career Regret on the AAMC GQ

Y2Q survey	Burnout at year 4^a^				Empathy at year 4^b^		Career choice regret at year 4^c^		
	Exhaustion coefficient (95% CI)	*P* value	Disengagement coefficient (95% CI)	*P* value	Coefficient (95% CI)	*P* value	Present, No. (%) (n = 989)	Absent, No. (%) (n = 13 097)	*P* value
Mistreatment									
Never	1 [Reference]	NA	1 [Reference]	NA	1 [Reference]	NA	689 (69.9)	10 135 (77.7)	<.001
Once	0.97 (0.76 to 1.18)	<.001	0.20 (0.07 to 0.33)	.003	0.06 (−0.14 to 0.27)	.55	111 (11.3)	1482 (11.4)	
More than once	1.81 (1.60 to 2.02)	<.001	0.71 (0.58 to 0.84)	<.001	−0.01 (−0.22 to 0.20)	.92	186 (18.8)	1433 (11.0)	
MSLES subscale									
Faculty interactions	−0.32 (−0.34 to −0.30)	<.001	−0.20 (−0.21 to −0.19)	<.001	0.17 (0.15 to 0.20)	<.001	13.7 (3.5)	15.0 (3.0)	<.001
Emotional climate	−0.40 (−0.42 to −0.38)	<.001	−0.26 (−0.28 to −0.25)	<.001	0.13 (0.11 to 0.16)	<.001	7.9 (3.3)	9.5 (3.0)	<.001
Student-student interaction	−0.29 (−0.31 to −0.26)	<.001	−0.18 (−0.19 to −0.16)	<.001	0.15 (0.12 to 0.17)	<.001	14.0 (3.4)	15.3 (2.9)	<.001

Abbreviations: AAMC, American Association of Medical Colleges; GQ, Graduation Questionnaire; MSLES, Medical School Learning Environment Survey; NA, not applicable; Y2Q, Medical School Year 2 Questionnaire.

^a^ Exhaustion scores range from 0 to 24, with higher scores indicating higher levels of exhaustion; disengagement scores, from 0 to 15, with higher scores indicating higher levels of depersonalization.

^b^ Scores range from 0 to 32, with higher scores indicating higher levels of empathy.

^c^ Missing numbers are excluded from the denominators.

**Figure.**
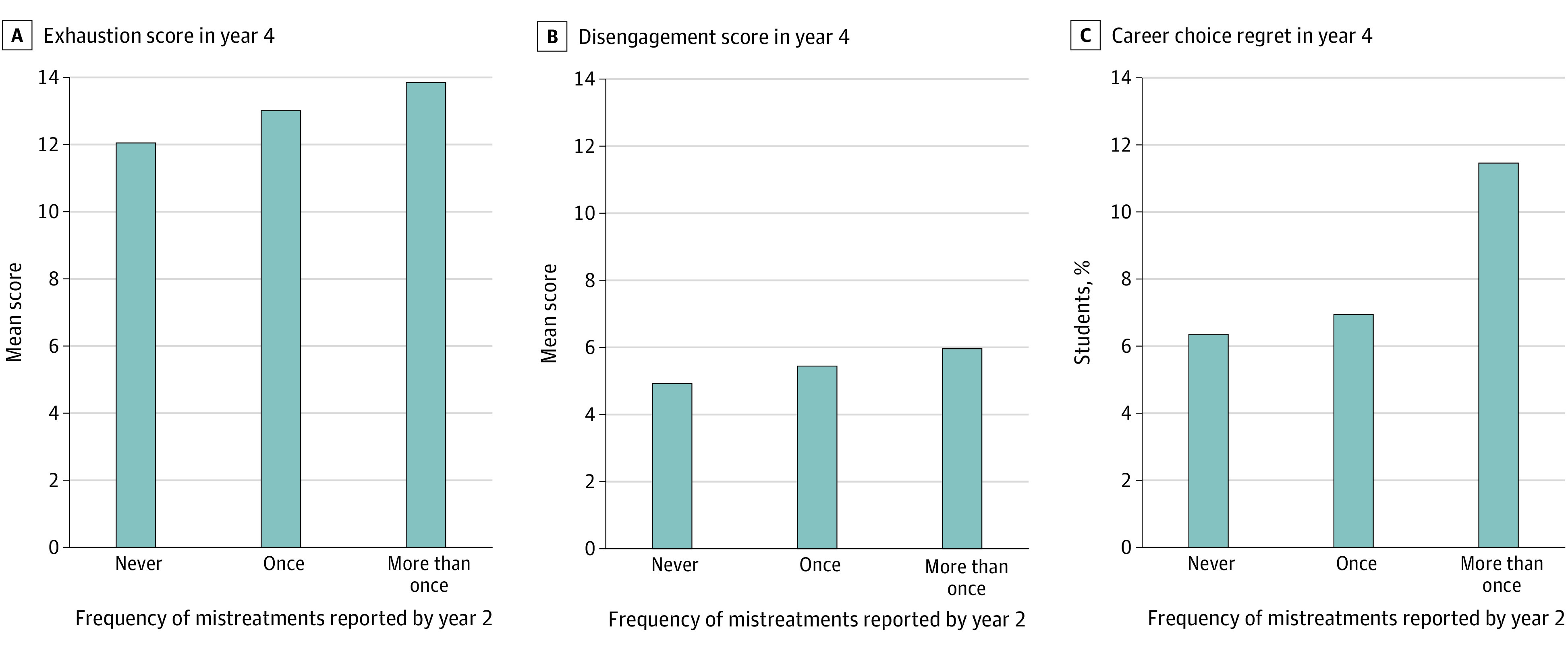
Association Between Frequency of Mistreatment Reported on the Association of American Medical Colleges (AAMC) Medical School Year 2 Questionnaire and the Exhaustion and Disengagement Scores and Career Regret on the AAMC Graduation Questionnaire Mistreatment occasionally or frequently was considered more than once. Exhaustion scores range from 0 to 24, with higher scores indicating higher levels of exhaustion; disengagement scores, from 0 to 15, with higher scores indicating higher levels of depersonalization.

### Multivariable Models for Burnout

After adjusting for Y2Q measures, mistreatment reported on the Y2Q was associated with a higher exhaustion score (once, 0.66 [95% CI, 0.51-0.81]; more than once, 1.74 [95% CI, 1.59-1.90]; overall *P* < .001) and a higher disengagement score (once, 0.29 [95% CI, 0.20-0.39]; more than once, 0.71 [95% CI, 0.61-0.81]; overall *P* < .001) on the GQ (Table 3). In contrast, perceptions of a more positive emotional climate reported on the Y2Q was associated with lower exhaustion score (for each 1-point increase, −0.05 [95% CI, −0.08 to −0.02]; *P* = .001) and lower disengagement score (for each 1-point increase, −0.04 [95% CI, −0.06 to −0.02]; *P* < .001) on the GQ.

**Table 3.** Multivariable Linear Regression for Burnout at End of Medical School Among US Medical Students Who Completed the AAMC Y2Q and the AAMC GQ

Variables reported at year 2 (Y2Q)	Exhaustion at year 4 (GQ)			Disengagement at year 4 (GQ)		
	Coefficient (95% CI)	*P* value	Overall *P* value	Coefficient (95% CI)	*P* value	Overall *P* value
No. of mistreatments						
0	1 [Reference]	NA	<.001	1 [Reference]	NA	<.001
1	0.66 (0.51 to 0.81)	<.001		0.29 (0.20 to 0.39)	<.001	
>1	1.74 (1.59 to 1.90)	<.001		0.71 (0.61 to 0.81)	<.001	
MSLES subscale (for each 1-point increase)						
Faculty interactions	−0.01 (−0.03 to 0.02)	NA	.69	0 (−0.02 to 0.02)	NA	.98
Emotional climate	−0.05 (−0.08 to −0.02)	NA	.001	−0.04 (−0.06 to −0.02)	NA	<.001
Student-student interactions	−0.02 (−0.05 to 0.00)	NA	.05	0 (−0.02 to 0.01)	NA	.58
Burnout (exhaustion or disengagement, for each 1-point increase)^a^	0.42 (0.40 to 0.44)	NA	<.001	0.48 (0.46 to 0.50)	NA	<.001
Empathy (for each 1-point increase)^b^	0.01 (−0.01 to 0.02)	NA	.52	−0.03 (−0.04 to −0.02)	NA	<.001
Overall QOL (for each 1-point increase)^c^	−0.03 (−0.07 to 0.02)	NA	.21	−0.02 (−0.04 to 0.01)	NA	.26
Stress (for each 1-point increase)^d^	0.12 (0.10 to 0.15)	NA	<.001	0 (−0.02 to 0.01)	NA	.56
Sex						
Female	−0.27 (−0.40 to −0.15)	NA	<.001	−0.47 (−0.55 to −0.39)	NA	<.001
Male	1 [Reference]	NA		1 [Reference]	NA	
Age (for each year older)	−0.02 (−0.04 to 0.01)	NA	.08	0.02 (0.01 to 0.03)	NA	.01
Marital status						
Single	1 [Reference]	NA	.10	1 [Reference]	NA	.65
Legally married	−0.17 (−0.33 to −0.02)	.03		−0.03 (−0.13 to 0.07)	.57	
Common law or civil union	0.48 (−0.46 to 1.42)	.32		0.42 (−0.18 to 1.01)	.17	
Divorced	0.36 (−0.26 to 0.98)	.25		0.17 (−0.22 to 0.56)	.40	
Separated but still legally married	−0.44 (−1.82 to 0.95)	.54		−0.14 (−1.02 to 0.73)	.75	
Widowed	−1.85 (−5.67 to 1.97)	.34		−0.47 (−2.88 to 1.95)	.70	
No. of dependents						
0	1 [Reference]	NA	.45	1 [Reference]	NA	.45
1	−0.15 (−0.43 to 0.14)	.30		0 (−0.18 to 0.18)	.97	
≥2	−0.17 (−0.51 to 0.18)	.35		−0.16 (−0.38 to 0.05)	.14	

Abbreviations: AAMC, American Association of Medical Colleges; GQ, Graduation Questionnaire; MSLES, Medical School Learning Environment Survey; QOL, quality of life; Y2Q, Medical School Year 2 Questionnaire.

^a^ As measured by a modified version of the Oldenburg Burnout Inventory (see Methods). Score ranges 8 to 32 for exhaustion and 5 to 20 for disengagement, with higher scores indicating higher levels of burnout.

^b^ As measured by the Interpersonal Reactivity Index scale. Scores range from 0 to 32, with higher scores indicating higher levels of empathy.

^c^ As measured by the single-item linear analogue QOL scale. Scores range from 0 to 10, with higher scores indicating better overall quality of life.

^d^ As measured by a 4-item Perceived Stress Scale. Scores range from 0 to 16, higher scores indicating higher perceived levels of stress.

A higher empathy score reported on the Y2Q was also associated with a lower disengagement score on the GQ (for each 1-point increase, −0.03 [95% CI, −0.04 to −0.02]; *P* < .001). A higher stress score reported on the Y2Q was associated with a higher exhaustion score on the GQ (for each 1-point increase, 0.12 [95% CI, 0.10-0.15]; *P* < .001). As expected, higher exhaustion and disengagement scores reported on the Y2Q were independently associated with higher exhaustion (0.42 [95% CI, 0.40-0.44]) and disengagement (0.48 [955 CI, 0.46-0.50]) scores on the GQ (Table 3). Compared with men, women had lower exhaustion (−0.27 [95% CI, −0.40 to −0.15]) and disengagement (−0.47 [95% CI, −0.55 to −0.39]) scores on the GQ, and older medical students had higher disengagement scores on the GQ (0.02 [95% CI, 0.01-0.03]) (Table 3). The overall *R^2^* values for the exhaustion and disengagement models were 0.31 and 0.29, respectively.

### Multivariable Models for Empathy

More positive student-faculty interactions as reported on the Y2Q MSLES faculty interactions subscale, but not mistreatment, were associated with a higher empathy score on the GQ (for each 1-point increase, 0.02 [95% CI, 0.001-0.05]; *P* = .04) after controlling for Y2Q measures (Table 4). In the multivariable model, a higher disengagement score on the Y2Q was also associated with a lower empathy score on the GQ (for each 1-point increase, −0.03 [95% CI, −0.06 to −0.004]; *P* = .03), whereas a higher empathy score on the Y2Q was associated with a higher empathy score on the GQ (for each 1-point increase, 0.63 [95% CI, 0.62-0.65]; *P* < .001). Compared with men, women (0.74 [95% CI, 0.63-0.85]; *P* < .001) and older medical students (0.05 [95% CI, 0.03-0.07]; *P* < .001) also had higher empathy scores (Table 4). The overall *R*^2^ value for the empathy model was 0.43.

**Table 4.** Multivariable Regression for Empathy and Career Regret at End of Medical School Among US Medical Students Who Completed the AAMC Y2Q and AAMC GQ

Variables reported at year 2 (Y2Q)	Empathy at year 4 (GQ)			Career regret at year 4 (GQ)		
	Coefficient (95% CI)	*P* value	Overall *P* value	Odds Ratio (95% CI)	*P* value	Overall *P* value
No. of mistreatments						
0	1 [Reference]	NA	.86	1 [Reference]	NA	<.001
1	0.01 (−0.112 0.15)	.83		1.35 (1.12 to 1.63)	.002	
>1	−0.03 (−0.17 to 0.11)	.67		1.87 (1.56 to 2.23)	<.001	
MSLES subscale (for each 1 point higher)						
Faculty interactions	0.02 (0.02 to 0.05)	NA	.04	1.02 (0.99 to 1.05)	NA	.32
Emotional climate	−0.02 (−0.05 to 0.01)	NA	.08	1.02 (0.98 to 1.05)	NA	.38
Student-student interactions	0 (−0.02 to 0.02)	NA	.86	0.97 (0.95 to 1.00)	NA	.04
Burnout^a^						
Disengagement (for each 1-point increase)	−0.03 (−0.06 to −0.004)	NA	.03	1.15 (1.11 to 1.20)	NA	<.001
Exhaustion (for each 1-point increase)	0.01 (−0.01 to 0.03)	NA	.22	1.07 (1.04 to 1.09)	NA	<.001
Empathy (for each 1-point increase)^b^	0.63 (0.62 to 0.65)	NA	<.001	0.98 (0.96 to 1.00)	NA	.03
Overall QOL (for each 1-point increase)^c^	0.02 (−0.01 to 0.0)	NA	.22	0.95 (0.90 to 0.99)	NA	.02
Stress (for each 1-point increase)^d^	0.01 (−0.01 to 0.04)	NA	.28	1.01 (0.97 to 1.04)	NA	.71
Career regret	NA	NA	NA	5.71 (4.60 to 7.10)	NA	<.001
Sex						
Female	0.74 (0.63 to 0.85)	NA	<.001	1.00 (0.98 to 1.03)	NA	.82
Male	1 [Reference]	NA		1 [Reference]	NA	
Age (for each year older)	0.05 (0.03 to 0.07)	NA	<.001	1.00 (0.97 to 1.02)	NA	.82
Marital status						
Single	1 [Reference]	NA	.85	1 [Reference]	NA	.90
Legally married	0.01 (−0.13 to 0.14)	.94		1.11 (0.92 to 1.34)	.28	
Common law or civil union	0.1 (−0.73 to 0.93)	.81		1.44 (0.51 to 4.07)	.49	
Divorced	0.01 (−0.56 to 0.57)	.98		1.00 (0.45 to 2.25)	>.99	
Separated, but still legally married	0.64 (−0.59 to 1.87)	.31		1.25 (0.26 to 5.99)	.78	
Widowed	2.02 (−2.13 to 6.16)	.34		NA	NA	
No. of dependents						
0	1 [Reference]	NA	.91	1 [Reference]	NA	.33
1	−0.05 (−0.31 to 0.20)	.69		1.19 (0.85 to 1.66)	.31	
2 or more	−0.04 (−0.35 to 0.27)	.79		0.82 (0.52 to 1.29)	.39	

Abbreviations: AAMC, American Association of Medical Colleges; GQ, Graduation Questionnaire; MSLES, Medical School Learning Environment Survey; NA, not applicable; QOL, quality of life; Y2Q, Medical School Year 2 Questionnaire.

^a^ As measured by a modified version of the Oldenburg Burnout Inventory (see Methods). Score ranges 8 to 32 for exhaustion and 5 to 20 for disengagement, with higher scores indicating higher levels of burnout.

^b^ As measured by the Interpersonal Reactivity Index scale. Scores range from 0 to 32, with higher scores indicating higher levels of empathy.

^c^ As measured by the single-item linear analogue QOL scale. Scores range from 0 to 10, with higher scores indicating better overall quality of life.

^d^ As measured by a 4-item Perceived Stress Scale. Scores range from 0 to 16, higher scores indicating higher perceived levels of stress.

### Multivariable Models for Career Regret

Mistreatment reported on the Y2Q was associated with higher odds of career regret on the GQ after controlling for Y2Q measures (odds ratio [OR] for once, 1.35 [95% CI, 1.12-1.63]; OR for more than once, 1.87 [95% CI, 1.56-2.23]; overall *P* < .001; Table 4). Better student-student interactions, as reported on the Y2Q MSLES student-student interactions subscale, were associated with lower odds of career regret on the GQ (OR for each 1-point increase, 0.97 [95% CI, 0.95-1.00]; *P* = .04). Higher exhaustion (OR for each 1-point increase, 1.15 [95% CI, 1.11-1.20]; *P* < .001) and disengagement scores (OR for each 1-point increase, 1.07 [95% CI, 1.04-1.09]; *P* < .001) on the Y2Q were also associated with higher odds of career regret on the GQ. In contrast, higher empathy score (OR for each 1-point increase, 0.98 [95% CI, 0.96-1.00]; *P* = .03) and QOL score (OR for each 1-point increase, 0.95 [95% CI, 0.90-0.99]; *P* = .02) on the Y2Q were independently associated with lower odds of career regret on the GQ.

## Discussion

Among medical students in this large national sample, those who experienced mistreatment and who perceived the learning environment less favorably were more likely to develop higher levels of exhaustion and disengagement, lower levels of empathy, and career regret compared with medical students with more positive experiences. These findings suggest the prevalence of burnout among medical students^8^ and students’ empathetic orientation and career satisfaction are, at least partially, attributable to factors within the learning environment.

In this cohort, 3224 of 14 076 respondents (22.9%) experienced mistreatment by the beginning of the second year of medical school. Although previous studies of learners have reported associations between mistreatment and burnout,^17,18,19,20^ ours is the first, to our knowledge, to be longitudinal in design and to explore potential associations with empathy and career choice regret. If we extrapolate our data to the full population of approximately 20 000 medical students, 2320 medical students are likely to experience being mistreated more than once by the beginning of year 2 of medical school. Among these students, we estimate that 11.3% (OR, 1.87 [95% CI, 1.56-2.23]) from the multivariable model) (Table 4) would experience career regret compared with 6.4% of students who did not experience mistreatment more than once. The increased risk of 4.9% means that 980 additional students may experience career regret owing to multiple mistreatment experiences with associated potential effects on well-being.

The potential protective effect of positive experiences within the learning environment may provide insight into strengths that organizations can amplify to mitigate burnout, decline in empathy, and career choice regret among their students. We found associations between measures of social and organizational components of the learning environment and student burnout, empathy, and career regret. Students’ perceptions of academic and nonacademic support and nurturing characteristics of faculty (eg, helpful when seeking advice or struggling academically, effective at providing feedback, approachable, and friendly) related to their subsequent levels of empathy. Students’ perceptions of social and academic support from peers were related to their career regret years later. How educational experiences made the student feel in terms of self-valuation, achievement, and confidence (ie, the emotional climate) was associated with their subsequent level of exhaustion and disengagement. These affective domains relate to individuals’ sense of self-efficacy, which has been shown to be an important motivational factor for learning that can be fostered by specific instructional strategies.^44^

At the end of medical school, female medical students had lower emotional exhaustion and depersonalization scores and higher empathy scores than male medical students after adjusting for mistreatment, perceptions of the learning environment, baseline scores across these domains, and demographics (age, marital status, and number of dependents). Other longitudinal studies exploring the prevalence of burnout by sex among medical students and residents have reported conflicting findings.^12,20,45,46,47^ Notably, in a prior study of surgical residents, female residents were more likely to have burnout, but this difference resolved after adjusting for mistreatment.^20^ However, in a longitudinal multispecialty cohort of US resident physicians,^47^ female residents were more likely to develop burnout and have worsening in the severity of their emotional exhaustion between the second and third year of training compared with male residents, even after controlling for various forms of mistreatment. Others have called for additional epidemiological research to better define risk factors for burnout among groups of learners, with special attention to sex and marginalized groups.^8^

These findings also point to potential interventions. Although the most effective approaches to addressing mistreatment of learners remain elusive,^48^ the frequency of mistreatment varies between educational programs,^20^ suggesting there are likely to be levers within the control of the organization that adequate commitment, leadership, infrastructure, resources, and accountability can lead to a meaningful reduction in mistreatment.^49^ Similarly, in a previous study of more than 4500 medical students attending 28 medical schools, the medical school campus explained the largest difference in MSLES scores.^50^ Strategies such as learning communities, pass/fail grading, and faculty development^44,50,51,52^ may help foster more positive learning environments. For example, pass/fail grading during the preclinical years has been shown to be associated with better group cohesion and lower stress levels among students without a detrimental effect on subsequent academic performance.^51,53,54^ Our study further suggests that lower stress levels at the beginning of year 2 of medical school may lessen the gravity of burnout symptoms during the clinical years. Furthermore, our finding that student-faculty interactions related to subsequent levels of empathy suggests that innovations to bolster empathy among medical students should go beyond communication skills training and other curricular approaches^55,56^ to include faculty development and improvement in system-level factors that hinder faculty prioritizing medical students’ education.^16,22^

### Limitations

Our study has limitations. First, the Y2Q and GQ included abbreviated measures (IRI and MSLES) and a modified version of the OBI, which we modified further after exploratory factor analysis and confirmatory factor analysis to improve the fit (in terms of internal validity) at the GQ time. The minimally important differences for exhaustion, disengagement, and empathy scale measures used have not been established. Additional work on validity is needed to better understand the construct being measured. However, the faculty interactions, emotional climate, and student-student interaction subscales of the MLES have been shown to be associated with subsequent US Medical Licensing Examination Step 1 scores, with each 1-point increase in subscale score associated with a nearly 3- to 7-point increase in the Step 1 score.^57^ The association between empathy and subsequent burnout (as measured by subscales from the Jefferson Scale of Physician Empathy and 2 single items from the Maslach Burnout Inventory, respectively) was also reported in a national longitudinal study of US residents.^23^ In addition, Cronbach α values were good to very good for the measures used, suggesting acceptable internal consistency or reliability.

Second, although the Y2Q survey contained several possible factors that are likely relevant, many were not measured, including academic performance, personal life events, moral distress, supervising resident behaviors, educational debt, and factors that may have been present before medical school matriculation. Third, the response rate to the Y2Q was estimated at 55.5%. The GQ survey response rate was estimated to be substantially higher at 81.5%, but in aggregate the final cohort represents approximately one-quarter of all medical students who graduated from 2016 to 2018. Although the sex distribution of our sample was similar to that of the full US medical student population, there may be important differences in the experiences of medical students who complete both AAMC surveys, and we are limited in our ability to make comparisons between students who chose to complete these surveys vs those who did not. We do not know the generalizability of the findings to all US medical students. Fourth, although our study is longitudinal, we cannot determine direction of effect definitively. However, there is consensus, as delineated in the National Academy of Medicine consensus study on burnout and a robust body of literature, that burnout is primarily a system-driven issue.^8^ Additional research is needed to determine student- and school-level interventions most likely to improve student well-being, empathy, and career satisfaction. Last, we did not have an identifying variable for each school in the analysis. Further analysis of school-level factors is a necessary next step to further clarify variability in learning environments that is based on institutional differences. Such variability might also be associated with the specific students within each school, rather than being solely attributed to the schools’ environments.

## Conclusions

Medical students who experienced mistreatment and had a less favorable perception of their learning environment were more likely to subsequently develop higher levels of exhaustion and disengagement, lower levels of empathy, and career regret compared with medical students with more positive experiences. Our findings suggest that strategies to improve student well-being, empathy, and experience should include approaches to eliminate mistreatment, optimize faculty-student interactions, build peer support, and enhance students’ self-efficacy.

## Notes

Supplement. **eTable.** Exploratory Factor Analysis and Confirmatory Factor Analysis for the Oldenburg Burnout Inventory and Graduation Questionnaire
