# Supplementary material for: Association of Characteristics of the Learning Environment and US Medical Student Burnout, Empathy, and Career Regret
Source: JAMA Netw Open. 2021 Aug 9;4(8):e2119110. doi: 10.1001/jamanetworkopen.2021.19110 (PMC8353540; doi:10.1001/jamanetworkopen.2021.19110)
Supplement: Supplement. — eTable. Exploratory Factor Analysis and Confirmatory Factor Analysis for the Oldenburg Burnout Inventory and Graduation Questionnaire [file jamanetwopen-e2119110-s001.pdf]

Supplementary Online Content

Dyrbye LN, Satele D, West CP. Association of characteristics of the learning environment and US medical student burnout, empathy, and career regret. *JAMA Netw Open*. 2021;4(8):e2119110. doi:10.1001/jamanetworkopen.2021.19110

eTable. Exploratory Factor Analysis and Confirmatory Factor Analysis for the Oldenburg Burnout Inventory and Graduation Questionnaire

This supplementary material has been provided by the authors to give readers additional information about their work.

© 2021 Dyrbye LN et al. *JAMA Network Open*.

eTable. Exploratory Factor Analysis and Confirmatory Factor Analysis for the Oldenburg Burnout Inventory and Graduation Questionnaire

Exploratory Factor Analysis (N=21,293)					
	**Q#**	**Description**	**F1**	**F2**	**F3**
Exhaustion	2	Tired before arriving to medical school	**0.60**	0.10	0.20
	3	Talk negative about medical school	**0.73**	0.52	0.29
	4	Need more time to relax	**0.73**	0.25	0.46
	6	Think less at medical school	**0.62**	0.42	-0.002
	8	Emotionally drained from medical school work	**0.73**	0.22	0.50
	9	Disconnected from medical school work	**0.61**	0.37	0.02
	11	Sickened by medical school work	**0.64**	0.30	0.39
	12	Feel worn out and weary	**0.71**	0.22	0.51
Disengage ment	1	Interesting aspects in work	0.29	**0.73**	0.21
	7	Medical School is a positive challenge	0.43	**0.73**	0.43
	13	Can only imagine studying medicine	0.03	**0.52**	0.12
	15	Engaged with medical school work	0.42	**0.79**	0.33
	16	Energized at medical school	0.47	**0.69**	0.43
Not included	5	Tolerate pressure of medical school	0.32	0.33	**0.80**
	10	Energy for leisure activities	0.43	0.23	**0.66**
	14	Manage medical school work	0.20	0.38	**0.76**

Confirmatory Factor Analysis with Correlated Factors (N=21,222)		
**Fit Summary**	**GQ Observed 3-factor Structure**	**Established 2-factor Structure**
Chi-square P	<0.0001	<0.0001
RMSEA Estimate (95% CI)	0.067 (0.066-0.068)	0.098 (0.097-0.099)
Bentler CFI	0.91	0.80
Bentler-Bonett NFI	0.91	0.80
Bollen Normed Index Rho1	0.89	0.77
James et al. Parsimonious NFI	0.77	0.69
Adjusted GFI	0.92	0.81
**Fit Summary**	**Y2 Observed 3-factor Structure**	**Established 2-factor Structure**
Chi-square P	<0.0001	<0.0001
RMSEA Estimate (95% CI)	0.064 (0.063-0.066)	0.091 (0.090-0.093)
Bentler CFI	0.92	0.84
Bentler-Bonett NFI	0.92	0.84
Bollen Normed Index Rho1	0.90	0.81
James et al. Parsimonious NFI	0.77	0.72
Adjusted GFI	0.93	0.84

© 2021 Dyrbye LN et al. *JAMA Network Open*.

We randomly divided the Y2Q and GQ survey responders into a development (Y2Q n = 14727; GQ n = 21293) and validation cohort (Y2Q n = 14733; GQ n = 21222). We conducted exploratory factor analysis on each development cohort followed by confirmatory factor analysis in the validation cohort. We included OBI items 2, 3, 4, 6, 8, 9, 11, and 12 in the exhaustion subscale and OLBI items 1, 7, 13, 15, and 16 in the disengagement subscale for both the Y2Q and GQ surveys as these items provided the best fit at the GQ time-point (dependent variable). Three items were eliminated as they did not load on either subscale. The resulting OBI exhaustion subscale had a Cronbach alpha of 0.81 (Y2Q) and 0.83 (GQ) and the disengagement subscale had a Cronbach alpha of 0.77 (Y2Q) and 0.72 (GQ). The possible range of scores was 0 to 24 and 0 to 15 for the exhaustion and personalization subscales, respectively, with higher scores equating to higher levels of exhaustion and disengagement.

© 2021 Dyrbye LN et al. *JAMA Network Open*.
